# B-Cell-Epitope-Based Fluorescent Quantum Dot Biosensors for SARS-CoV-2 Enable Highly Sensitive COVID-19 Antibody Detection

**DOI:** 10.3390/v14051031

**Published:** 2022-05-12

**Authors:** Yucheng Zheng, Kun Song, Kun Cai, Linlin Liu, Dixiao Tang, Wenbo Long, Bohui Zhai, Jianjun Chen, Yanbing Tao, Yunong Zhao, Simeng Liang, Qing Huang, Qianyun Liu, Qi Zhang, Yu Chen, Yingle Liu, Huayao Li, Ping Wang, Ke Lan, Huan Liu, Ke Xu

**Affiliations:** 1 State Key Laboratory of Virology, Institute for Vaccine Research, College of Life Sciences, Wuhan University, Wuhan 430072, China; 2020102040034@whu.edu.cn (Y.Z.); kunsong@whu.edu.cn (K.S.); sane0909@whu.edu.cn (D.T.); 2015301060039@whu.edu.cn (S.L.); liuqianyun@whu.edu.cn (Q.L.); gracetey@whu.edu.cn (Q.Z.); chenyu@whu.edu.cn (Y.C.); mvlwu@whu.edu.cn (Y.L.); klan@whu.edu.cn (K.L.); 2 Hubei Provincial Center for Diseases Control and Prevention, Wuhan 430079, China; ckreal@163.com (K.C.); mice0809@163.com (L.L.); 3 School of Optical and Electronic Information, Wuhan National Laboratory for Optoelectronics, Optics Valley Laboratory, Huazhong University of Science and Technology, 1037 Luoyu Road, Wuhan 430074, China; longwenbo@hust.edu.cn (W.L.); bohui_zhai@hust.edu.cn (B.Z.); taoyanbing@hust.edu.cn (Y.T.); zyn248@hust.edu.cn (Y.Z.); huangqingcl@163.com (Q.H.); huayaoli@hust.edu.cn (H.L.); 4 Department of Otorhinolaryngology, Union Hospital, Tongji Medical College, Huazhong University of Science and Technology, 1277 Jiefang Avenue, Wuhan 430022, China; ylly80331@163.com; 5 Institute for Vaccine Research, Animal Biosafety Level 3 Laboratory, Wuhan University, Wuhan 430072, China; 6 Department of Clinical Laboratory, Union Hospital, Tongji Medical College, Huazhong University of Science and Technology, 1277 Jiefang Avenue, Wuhan 430022, China; wping7722@sina.com

**Keywords:** SARS-CoV-2 diagnosis, nanometer-scale fluorescent biosensors, B-cell epitopes, quantum dots

## Abstract

A new antibody diagnostic assay with more rapid and robust properties is demanded to quantitatively evaluate anti-severe acute respiratory syndrome coronavirus 2 (SARS-CoV-2) immunity in a large population. Here, we developed a nanometer-scale fluorescent biosensor system consisting of CdSe-ZnS quantum dots (QDs) coupled with the highly sensitive B-cell epitopes of SARS-CoV-2 that could remarkably identify the corresponding antibody with a detection limit of 100 pM. Intriguingly, we found that fluorescence quenching of QDs was stimulated more obviously when coupled with peptides than the corresponding proteins, indicating that the energy transfer between QDs and peptides was more effective. Compared to the traditional enzyme-linked immunosorbent assay (ELISA), the B-cell-epitope-based QD-biosensor could robustly distinguish coronavirus disease 2019 (COVID-19) antibody-positive patients from uninfected individuals with a higher sensitivity (92.3–98.1% positive rates by QD-biosensor vs. 78.3–83.1% positive rates by ELISAs in 207 COVID-19 patients’ sera) in a more rapid (5 min) and labor-saving manner. Taken together, the ‘QD-peptides’ biosensor provided a novel real-time, quantitative, and high-throughput method for clinical diagnosis and home-use tests.

## 1. Introduction

The first coronavirus disease 2019 (COVID-19) case was reported at the end of 2019 in Wuhan, China [1]. There have been over 511 million reported cases and 6.2 million deaths globally (https://covid19.who.int/, accessed on 3 May 2021). It is widely accepted that accurate, rapid, and reliable diagnostics are essential tools to help control the pandemic [2]. The standard method for the COVID-19 diagnosis is the detection of viral nucleic acids by RT-PCR, which can be applied quickly as soon as the virus sequence is disclosed. However, the possibilities of false-negative test results due to differences in specimen source [3] and time of sample collection [2] limit the credibility of the RT-PCR results. The time-consuming testing process also restricts the rapid use of the RT-PCR diagnostic method [4]. Another prevalent diagnostic method, serological tests, which can help to monitor the severe acute respiratory syndrome coronavirus 2 (SARS-CoV-2)-specific antibody level in patients, combined with RT-PCR, can further improve the accuracy of the diagnosis [5]. Clinically, enzyme-linked immunosorbent assay (ELISA) methods have been used on a large scale to monitor the titers of SARS-CoV-2-specific antibodies in the sera of COVID-19 patients or the vaccinated population [6]. Unfortunately, ELISA is a cumbersome, laboratory-based technique that relies on equipment and specialized personnel that requires hours to deliver a result [7]. Thus, a more robust, fast, and quantitative serological test method can help evaluate the infection rate and herd immunity in a large population [8].

The SARS-CoV-2 virion is composed of four main structural proteins: spike (S) proteins, nucleocapsid (N) proteins, membrane (M) proteins, and envelope (E) proteins [9]. The recombinant S proteins and N proteins are the preferential antigens for serological assays [10]. However, full-length S and N proteins as diagnostic antigens may result in cross-reactivity, causing false-positive results due to pre-existing antibodies against other human coronaviruses, particularly for four common cold-causing coronaviruses HCoV-OC43, HKU1, NL63, and 229E [11,12]. Therefore, it is necessary to identify virus-specific epitopes that are highly immunogenic and less homologous to the N and S proteins of related coronaviruses to develop highly specific serological assays [13].

Compared with full-length proteins, epitopes are the targeted structures recognized by B cells to produce antibodies and are also the basis of the specificity of the immune response. The specificity can be significantly amplified by using epitopes as diagnostic antigens [13]. However, due to the small size of the peptides, a comparable carrier or medium to carry the peptides is a critical factor that determines the diagnostic quality [14]. Quantum dots (QDs) are inorganic semiconductor nanomaterials with high quantum efficiency, good photochemical stability, and controllable fluorescence properties [15]. Compared with conventional fluorescent dyes (<5 ns), such as SYBR Green, QDs have a high photobleaching resistance (100- to 1000-fold) and a longer excited-state lifetime (10–40 ns) [16]. QDs can be easily modified by biomolecules, allowing them to be a qualified carrier for various substrates [17,18]. Fluorescent QDs and peptides can match each other’s size on the nanometer scale and have been applied to a variety of biological detections; for example, QDs with cell-penetrating peptides can monitor lysosomal pH fluctuations [19], QDs with histone-containing peptides can mark *E. coli* [20], and QDs with glutathione can detect levodopa concentrations in vivo [21]. Nevertheless, whether QDs can be a rapid and sensitive detection method for the SARS-CoV-2 diagnosis is unknown.

This study fabricated a fluorescent QD-peptides biosensor coupled with particular SARS-CoV-2 B-cell epitopes to speed up the diagnosis and evaluate immunity in a large population on site. With this in mind, we screened the highly sensitive SARS-CoV-2 peptides by conformational and linear B-cell epitope predictions. The resulting high-affinity B-cell epitopes were conjugated with characterized QDs to form QD-peptides that could detect specific binding from anti-SARS-CoV-2 antibodies more rapidly, quantitatively, and automatically.

## 2. Materials and Methods

### 2.1. Materials and Chemicals

The carboxyl-based water-soluble QDs obtained by modifying the surface of hydrophobic polyacrylic acid were bought from Wuhan Jiayuan Quantum Dots Co., Ltd. (Wuhan, China). The anti-SARS-CoV-2 Spike RBD polyclonal antibodies, the anti-MERS S_full_ polyclonal antibodies, the anti-HKU1 S_1-760_ polyclonal antibodies, the anti-SARS-CoV-2 N polyclonal antibodies, the anti-HKU1 N polyclonal antibodies, the anti-NL63 N polyclonal antibodies, the SARS-CoV-2 spike RBD recombinant proteins, and SARS-CoV-2 N recombinant proteins were purchased from Sino Biological (Beijing, China). 3,3′,5,5′-Tetramethylbenzidine (TMB) liquid substrate was purchased from NCM Biotech (Suzhou, China). Other chemicals and reagents were purchased from Sinopharm Chemical Reagent Corp., (Shanghai, China) unless otherwise noted.

### 2.2. Serum Samples

Serum samples were collected from 210 SARS-CoV-2 convalescents, 10 SARS-CoV-2 omicron-infected patients, 10 SARS-CoV-2 delta-infected patients, and 33 healthy individuals at the Hubei Provincial Centre For Disease Control and Prevention/Hubei Provincial Academy Of Preventive Medicine (HBCDC) with written consent under the appropriate institutional review boards’ approval (2021-012-01) and were identified. All patients provided written informed consent. All patient clinical records were listed in Tables S7 and S8.

All serum samples were heat-inactivated for 30 minutes at 56 °C.

### 2.3. Epitope Prediction and Polypeptide Synthesis

Prediction of conformational B-cell epitopes was performed by using the SARS-CoV-2 spike proteins’ structure (PDB: 6VSB) with DiscoTope 2.0 and a cutoff of ≥−2.5 (corresponding to 80% specificity). Linear B-cell epitope predictions were carried out on the spike proteins (NCBI: YP_009724390.1) and nucleocapsid proteins (NCBI: YP_009724397.2) with BepiPred 2.0. A cutoff of ≥0.5 was used to predict linear B-cell epitopes of the S and N proteins. Then, Emini Surface Accessibility Prediction and a cutoff of ≥1.0 were used to predict linear B-cell epitopes on the S and N proteins once again. Finally, epitope sequences, each consisting of 11–15 amino acids with high scores in the prediction method of the S proteins epitope and N proteins epitope, were identified.

The selected epitope sequences were then sent to GenScript (Nanjing, China) for commercial biotinylated synthesis. The peptides were dissolved in ddH_2_O, prepared as a stock solution at a concentration of 1 mg/mL, and stored at −20 °C.

### 2.4. Homologous Analysis and Proteins Model

Sequence alignments between the epitopes of coronaviruses were performed by using DNAMAN software. The following viral gene sequence information was obtained from the National Center for Biotechnology Information (NCBI) database: SARS-CoV-2 WIV04 spike proteins (GenBank: QHR63260.2), SARS-CoV-2 WIV04 nucleocapsid proteins (GenBank: QHR63268.1), SARS Tor2 spike proteins (GenBank: AAP41037.1), SARS Tor2 nucleocapsid proteins (GenBank: AAP41047.1), MERS spike proteins (GenBank: AZK15900.1), MERS nucleocapsid proteins (GenBank: AZK15907.1), human coronavirus 229E spike proteins (GenBank: ABB90529.1), human coronavirus 229E nucleocapsid proteins (GenBank: AGW80953.1), human coronavirus OC43 spike proteins (GenBank: QEG03814.1), human coronavirus OC43 nucleocapsid proteins (GenBank: AAT84366.1), human coronavirus NL63 spike proteins (GenBank: APF29063.1), human coronavirus NL63 nucleocapsid proteins (GenBank: AFV53152.1), human coronavirus HKU1 spike proteins (GenBank: AGW27881.1), and human coronavirus HKU1 nucleocapsid proteins (GenBank: ARU07581.1).

The structure of the SARS-CoV-2 spike open state (PDB: 6VYB) was visualized using PyMOL.

### 2.5. ELISA

The reaction between the antibody and peptide was confirmed by ELISA as described in previous reports [22] with minor changes. Briefly, standard 96-well plates (Costar 3590) were coated overnight at 37 °C with 100 µL of 50 µg/mL streptavidin diluted in 0.1 M carbonate buffer (pH = 9.6). Plates were subsequently washed three times with 0.05% PBST (0.05% Tween-20 in PBS). Excess binding sites were blocked for 2 h at 37 °C using 200 µL of 0.05% PBST containing 0.4% gelatin. Afterward, biotin-labeled peptides (1 mg/mL) were prepared in 0.4% gelatin at a 1:1000 dilution, and 100 µL was added per well at 37 °C for an incubation time of 2 h. The plates were then washed and blocked with 200 μL per well of 5% skim milk. Serial dilutions of serum samples were added at 37 °C for 2 h followed by the addition of the anti-human IgG-horseradish peroxidase (HRP)-conjugated secondary antibody for 1 h. The plates were again washed three times with 0.05% PBST, and the remaining peroxidase activity in the wells was assayed by the addition of 100 µL of TMB substrate in the dark at room temperature for 30 min. The reaction was stopped by adding 50 µL per well of 0.1 M HCl. The absorbance at 450 nm (OD_450_) was measured with a microplate reader. Protein-antibody reactions were detected using a similar method, except that the proteins (1 ng/µL) were coated directly in the 96-well plate rather than the plates being precoated with streptavidin.

### 2.6. Preparation of QDs Fluorescence Probes

An amount of 2 µL water-soluble QD-COOH solution (8 µM CdSe-ZnS) was added to 2.5 mL of phosphate buffer solution (0.0067 M, pH = 7.0–7.2), and then 2 µL of EDC solution (20 mg/mL N-(3-dimethylaminopropyl)-N’-ethylcarbodiimide hydrochloride in PBS) was added to the QDs phosphate buffer solution. A total of 10 µL of peptide or protein solution (10 ng/uL peptides or proteins in PBS) was added to the mixture, which was placed at room temperature for 8 h to promote coupling between the peptides or proteins and QDs. After peptides or proteins binding, excess peptide molecules or proteins were removed using a mini dialysis kit (GE Healthcare, Chicago, FL, USA). Finally, 2 µL of BSA (20 mg/mL albumin from bovine serum in PBS) was added to the mixture, and the mixture was placed at room temperature for another 8 h to block the QDs. Through the above steps, a solution of QDs coupled with peptides or proteins was obtained.

### 2.7. TEM

TEM instrumentation (Tecnai G2 F30, FEI Company, Hillsboro, OR, USA) was used to obtain the particle size and shape of the QDs and QDs coupled with S_epi1_. QDs or QD-S_epi1_ were prepared in PBS solution, and a drop of the solution was placed on a carbon-coated copper network and dried at room temperature for TEM imaging.

### 2.8. Absorption Spectrum Measurement

QDs and QD-S_epi1_ solutions were prepared in PBS solution, and the normalized absorption spectrum was measured with a spectrofluorometer (UV-3600 plus, Shimadzu, Beijing, China).

### 2.9. FTIR

QDs and QD-S_epi1_ solutions were prepared in PBS solution, dropped onto tin foil, and allowed to evaporate for approximately 1 h to form films. Each film was scanned 16 times with an FTIR spectrometer (Vertex 70V, Bruker, Billerica, MA, USA).

### 2.10. Detection of the Serum or Antibodies by Specific QDs

The fluorescence intensity of each solution was measured with a multifunctional microplate reader (Varioskan LUX, Thermo Scientific, Waltham, MA, USA). After adding 10 µL of diluted serum or different concentrations of antibodies to the QD-peptides, the mixtures were gently shaken and left for 5 min to determine the fluorescence intensity at different wavelengths. The fluorescence quenching strength was calculated by subtracting the fluorescence intensities of the QD-peptides in the presence (peak value Y2) from absence (peak value Y1) of the serum or the antibody at 608 nm. The fluorescence quenching ratio was defined as (peak value Y1 − peak value Y2) / peak value Y1 × 100%.

The fluorescence quenching ratio curve was generated with the fluorescence intensity of the solution measured every 30 s after 10 μL of the diluted antibody was added to the QD-peptides, and the corresponding fluorescence quenching ratio was calculated according to the above formula.

### 2.11. Statistical Analysis

Data were analyzed using GraphPad Prism version 8 and Microsoft Excel 2016. If not indicated otherwise, Student’s *t*-test and two-way ANOVA were used for two-group comparisons. Descriptive statistics analysis including dispersion coefficient was used for each data set. The Spearman’s rank coefficient measured the associations between ELISAs and the biosensors. * *p* < 0.05, ** *p* < 0.01, *** *p* < 0.001, and **** *p* < 0.0001 were considered significant.

## 3. Results

### 3.1. Characterization of the Fluorescent QD-Peptides Biosensor

**Figure 1:**
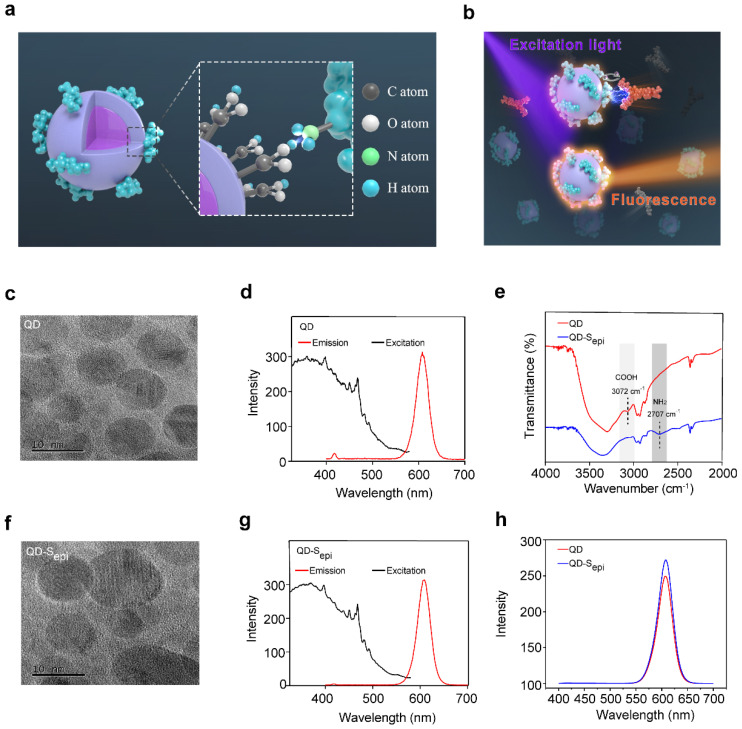
Schematic illustration of QD-peptides detection for the corresponding antibody. (**a**) Schematic of QDs coupled with peptides. The peptides are marked in cyan. (**b**) A schematic diagram of the fluorescent QD-peptides biosensor identifies antibodies by the quantitative fluorescent quenching signal. Purple represents excited light, and orange represents emitted light. TEM (**c**) and emission and excitation spectra (**d**) of the QDs. TEM (**f**) and emission and excitation spectra (**g**) of the QDs coupled with an epitope from receptor-binding-domain (RBD) of SARS-CoV-2 S proteins (S_epi_). (**e**) FTIR spectra of the QDs (red line) and QDs coupled with S_epi_ (blue line). The carboxyl peak (COOH; 3072 cm^−1^) is shown in light grey and the amino peak (NH_2_; 2707 cm^−1^) is in dark grey. (**h**) Fluorescence intensity of QDs (red line) and QDs coupled with S_epi_ (blue line). S_epi_: S_epi1_ (aa491–505).

To fabricate a high-sensitive biosensor, we used 1-ethyl-3-(3-dimethylaminopropyl) carbodiimide (EDC) to couple the carboxyl groups on the CdSe-ZnS core-shell QD surfaces with the amino groups of the SARS-CoV-2 peptides to form a fluorescent QD-peptides biosensor (coupling strategy shown in Figure 1a). We chose the CdSe-ZnS QDs in this study because of their high fluorescence efficiency [23,24,25]. Upon QDs coupled with peptides, the binding of the corresponding antibody might shade the emitted light so that a bioreaction between the antigen and the antibody could be quantitatively detected by the fluorescent quenching signal (working model shown in Figure 1b). To explore whether QDs coupled with peptides would change the physical properties of the QDs, the QDs were conjugated with an epitope (S_epi_) from receptor-binding-domain (RBD) of SARS-CoV-2 S proteins to form the QD-S_epi_ complex. The TEM images indicated that the QDs (Figure 1c) and QD-S_epi_ (Figure 1f) were both nanocrystals with a similar diameter of approximately 5~10 nm. Under the same excitation light at 365 nm, the emission spectra of the QDs (Figure 1d) and QD-S_epi_ (Figure 1g) showed the identical peak wavelength at 608 nm; the changes in their exciton patterns were neglectable. The results indicated that the QDs coupled with SARS-CoV-2 peptides maintained their inherent fluorescent characteristics. We further conducted the Fourier transform infrared spectroscopy (FTIR) measurement to determine the variation in chemical bonds after coupling QDs with SARS-CoV-2 peptides. The commercial CdSe-ZnS QDs underwent surface carboxylation treatment, showing an absorption peak at 3072 cm^−1^ (light grey) corresponding to the exposure of the COOH group (Figure 1e). By contrast, the 3072 cm^−1^ peak disappeared in the QD-S_epi_ complex, while a new absorption peak at 2707 cm^−1^ (dark grey) corresponding to the NH_2_ group appeared, indicating the successful link of the peptide (Figure 1e). Additionally, peptides conjugation to the carboxyl groups of QDs increased the fluorescence intensity from 243 to 278 at 608 nm, again indicating a successful link and energy transfer in the QD-peptides complex (Figure 1h). The enhanced QD-peptides fluorescence would further favor achieving a higher signal-to-noise ratio for antibody detection.

### 3.2. QD-SARS-CoV-2 Peptides React with the Corresponding Antibody

**Figure 2:**
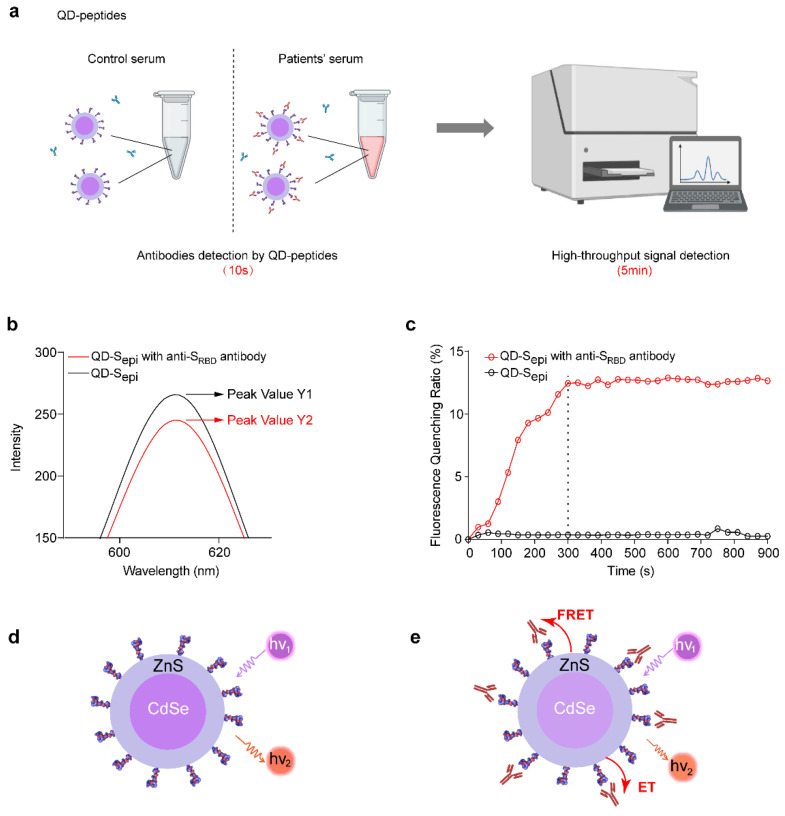
Model of QD-SARS-CoV-2 peptides reacting with the corresponding antibody. (**a**) Schematic diagram of COVID-19 patients’ sera detected by the QD-peptides biosensor. (**b**) Fluorescence intensity of QD-S_epi_ reacting with (red line, peak value Y2) or without (black line, peak value Y1) the anti-S_RBD_ antibody at 608 nm. The fluorescence quenching ratio was defined as (fluorescent peak value Y1 − fluorescent peak value Y2)/Y1 × 100%. (**c**) The time-resolved measurement results of the fluorescence quenching ratio for QD-S_epi_ with (red line) or without (black line) the anti-S_RBD_ antibody. The fluorescence quenching ratio becomes stable in 5 min (300 s) marked by the dashed line. Experiments were repeated three times with similar results. (**d**,**e**) The sensing mechanism of the QD-peptides biosensor reacting with the corresponding antibody. (**d**) Schematic diagram of the QD-peptides. (**e**) Schematic diagram of the QD-peptides reacting with the corresponding antibody. The dark purple area represents the CdSe core of the QDs, the light purple area represents the ZnS shell of the QDs, and the red area represents the antibody. ET, electron transfer. FRET, fluorescence resonance energy transfer. S_epi_: S_epi1_ (aa491–505).

The flow chart of antibody detection by the QD-peptides biosensor is shown in Figure 2a, wherein the high-throughput fluorescent signal detection could be achieved using a multifunctional microplate reader within 5 min. We first explored whether QDs coupled with SARS-CoV-2 peptides could be applied to detect commercial antibodies. Compared with QD-S_epi_ in the absence of antibodies (black line, Figure 2b), the fluorescence intensity was significantly reduced after being mixed with anti-S_RBD_ antibodies (red line, Figure 2b). The results indicated that fluorescence quenching of QDs occurred when S_epi_ was bound to the antibody. The fluorescence quenching ratio was defined as (fluorescent peak value Y1 of QD-S_epi_ without antibodies-fluorescent peak value Y2 of QD-S_epi_ with antibodies)/Y1 × 100%. The time-resolved measurement results further indicated that the fluorescence quenching ratio of QD-S_epi_ became stable after 5 min in the presence of anti-S_RBD_ antibodies (Figure 2c), suggesting the equilibrium of the reaction between peptides with antibodies. Therefore, a QD-peptides biosensor could be used to detect the corresponding antibody, and the reaction time of the whole detection process was only 5 min.

We further proposed a geometrical match hypothesis to explain the principle of our sensor design (sensing mechanism shown in Figure 2d–e). The double-layer heterostructure of the QDs (core: CdSe, shell: ZnS) ensured the formation of a highly stable type-I quantum well that favored the radiation recombination. When 365 nm excitation light excited the QDs, the valence band’s electrons were excited into the conduction band and returned to the valence band by emitting red light at 608 nm (Figure 2d). However, upon antibody binding to QD-peptides, electron transfer (ET) or fluorescence resonance energy transfer (FRET) between the antibody and QDs [26] may occur through peptide bridging, leading to reduced light emission at 608 nm (Figure 2e).

### 3.3. Identification of Four Highly Sensitive B Cell Epitopes of SARS-CoV-2

**Figure 3:**
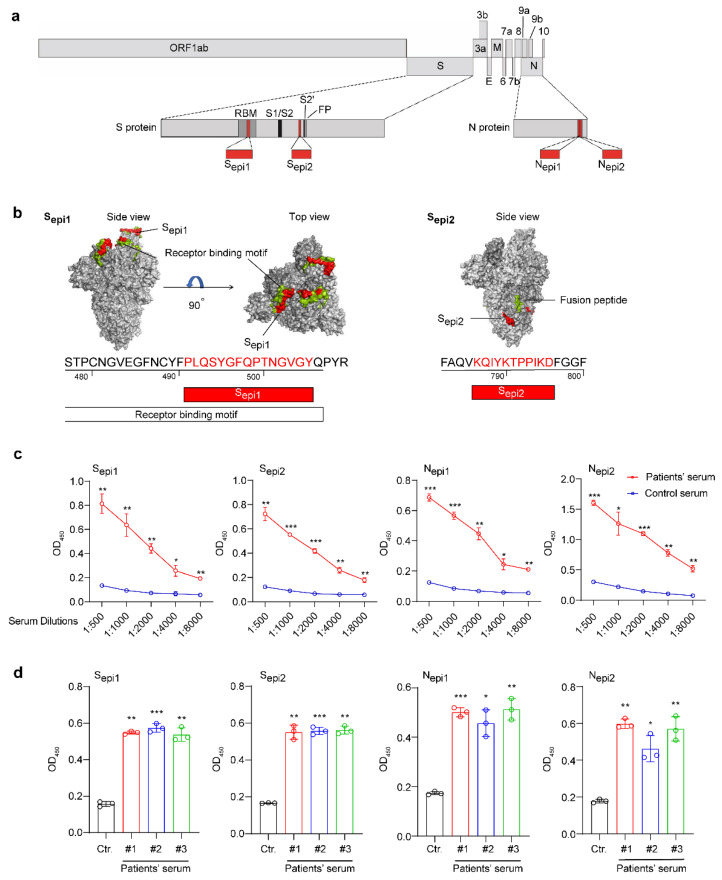
Identification of the antigenic epitopes of SARS-CoV-2 S and N proteins. (**a**) Schematic of the genomic structure of SARS-CoV-2. Different grey boxes represent different ORFs. The epitopes are marked in red. (**b**) Visualization of the trimeric S protein of SARS-CoV-2 based on PDB 6VYB using PyMOL. S_epi1_ and S_epi2_ are marked in red. Receptor binding motifs and fusion peptides are marked in cyan. (**c**) The reaction of a COVID-19 antibody-positive patient’s serum (red curve) and an uninfected human serum (blue curve) at different dilutions of the four peptides by ELISAs. (**d**) Reaction of 3 COVID-19 antibody-positive patients (#1, red; #2, blue; and #3 green) and uninfected human serum(black) to the four peptides by ELISA. The serum was diluted at 1:2000 (except for patient serum #1 tested with N_epi2_, which was diluted at 1:4000). All data are presented as the mean ± SD from triplicate measurements. All statistical analyses were carried out with unpaired *t*-tests for each experiment (* *p* < 0.05, ** *p* < 0.01, *** *p* < 0.001).

To find highly sensitive epitopes for QD-peptides biosensors, we screened the B-cell epitopes of the S and N proteins from SARS-CoV-2 by both conformational and linear B-cell epitope predictions (Figures S1 and S2 and Tables S2–S6). We found two high-ranking B-cell epitopes on the S proteins, S_epi1_ (aa491–505, PLQSYGFQPTNGVGY; located within the receptor-binding motif (RBM) presenting neutralizing epitopes) and S_epi2_ (aa786–796, KQIYKTPPIKD; located close to the fusion peptide, also present in the protein structure in Figure 3b), and two high-ranking B-cell epitopes of the N proteins, located adjacent to each other at the C-terminus of the N proteins, N_epi1_ (aa367–377, EPKKDKKKKAD) and N_epi2_ (aa380–390, QALPQRQKKQQ). All four peptides are linear in structure and the position of these four epitopes in the context of the SARS-CoV-2 genome is shown in Figure 3a.

To further assess the cross-reactivity, we explored the homology between these SARS-CoV-2 epitopes and other human coronaviruses [11] (229E, OC43, NL63, HKU1, severe acute respiratory syndrome coronavirus (SARS-CoV), and Middle East respiratory syndrome coronavirus (MERS-CoV)). Except for SARS-CoV (60–91% homology), the four epitopes showed little identity with MERS-CoV, HKU1, OC43, 229E, and NL63, which varied from 9–45%, 9–27%, 9–18%, 0–13%, and 0–7%, respectively (Table S1).

We further proved that the four SARS-CoV-2 peptides with high immunogenicity could be used to distinguish the sera of COVID-19 patients from those uninfected. First, we diluted serum (1:500–1:8000) to react with the four peptides and found that these peptides could significantly distinguish COVID-19 antibody-positive patients from uninfected individuals, even if the serum was diluted 8000-fold (Figure 3c). The sample was then expanded to three patients (patient serum #1, #2, and #3). Once again, the peptides could significantly distinguish the COVID-19 antibody-positive patients from uninfected individuals at a 1:2000 dilution (except for patient serum #1, which was tested with N_epi2_ diluted 1:4000) (Figure 3d). Therefore, these four peptides with high immunogenicity could be used to diagnose COVID-19 patients.

### 3.4. QDs Coupled with Four Highly Sensitive SARS-CoV-2 Peptides Efficiently Respond to the Corresponding Antibody

**Figure 4:**
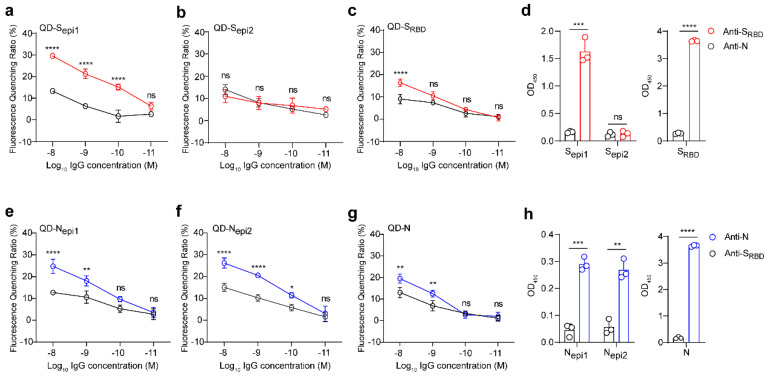
QDs coupled with four highly sensitive SARS-CoV-2 peptides efficiently respond to the corresponding antibody. (**a**–**c**) Reactivity of QDs coupled with S_epi1_ (**a**), S_epi2_ (**b**), and S_RBD_ (**c**) to the anti-S_RBD_ antibody (red) and anti-N antibody (black). (**d**) Reactivity of S_epi1_, S_epi2,_ and S_RBD_ to the anti-S_RBD_ antibody (red) and anti-N antibody (black) by ELISAs. (**e**–**g**) Reactivity of QDs coupled with N_epi1_ (**e**), N_epi2_ (**f**), and the recombinant N protein (**g**) to the anti-N antibody (blue) and anti-S_RBD_ antibody (black). (**h**) Reactivity of N_epi1_, N_epi2,_ and the recombinant N protein to anti-N antibody (blue) and anti-S_RBD_ antibody (black) by ELISAs. All values represent the means ± SD of three independent experiments. Statistical analyses were carried out with unpaired *t*-tests for ELISAs and two-way ANOVA for fluorescence quenching ratio comparisons. * *p*  <  0.05, **p  <  0.01, *** *p*  <  0.001, **** *p*  <  0.0001, ns, non-significant.

To verify the performance of these four QD-peptides biosensors, we evaluated the reactivity between S peptides (S_epi1_ and S_epi2_; S_RBD_ proteins as a control) and a commercial anti-S_RBD_ antibody (an anti-N antibody as a control) through either fluorescent QD-peptides biosensors or ELISAs. The QDs coupled with the S_epi1_ (located inside the S_RBD_ domain), had a strong reaction with anti-S_RBD_ antibodies but not with anti-N control antibodies, and the detection limit was 100 pM (Figure 4a, *p* < 0.0001). On the contrary, the QDs coupled with the S_epi2_ (located outside the S_RBD_ domain) did not react with either the anti-S_RBD_ or anti-N control antibodies (Figure 4b). However, to our surprise, the QDs coupled with S_RBD_ proteins only responded with high concentrations of anti-S_RBD_ antibodies (10 nM) (Figure 4c), indicating that QD-S_RBD_ could not work as efficiently as QD-S peptides for antibodies detection. However, in the traditional ELISAs, both S_RBD_ proteins and S_epi1_ reacted strongly with the anti-S_RBD_ antibody (Figure 4d). The results suggested that only the QD-S peptides but not the QD-S_RBD_ proteins could respond to the corresponding anti-S_RBD_ antibody at a low concentration.

Similarly, we evaluated the reactivity between N peptides (N_epi1_ and N_epi2_, N proteins as a control) and a commercial anti-N antibody (an anti-S_RBD_ antibody as a control) through either fluorescent QD-peptides biosensors or ELISAs. Both QD-peptides (N_epi1_ and N_epi2_) biosensors reacted strongly with 100 pM-10 nM anti-N antibodies (Figure 4e, *p* < 0.01 and 4f, *p* < 0.05). In contrast, the QD-N proteins only reacted with high concentrations (1 nM–10 nM) of the anti-N antibodies (Figure 4g), indicating that the QD-peptides worked more efficiently as biosensors than QD-proteins. As a control, all of the N proteins, N_epi1_, and N_epi2_ showed strong reactivities to the anti-N antibodies in the ELISAs (Figure 4h).

Although both proteins and peptides were successfully conjugated to QDs, their spatial structure and molecular characteristics differed. Proteins are macromolecules with a 3D structure, and peptides are small, long-chain organic molecules with linear structures. Thus, peptides may enable their sufficient and abundant presence on the QDs surface. Instead, the energy coupling between the QDs and antigen protein is weak due to their geometrical mismatch. Therefore, our work demonstrated the unique advantage of peptides over protein in the fluorescent QDs biosensors.

Of note, as the QD-peptides could respond to a low antibody concentration of 100 pM (fluorescent quenching ratio was 15.2 % for QD-S_epi1_, 9.6% for QD-N_epi1_, and 11.5% for QD-N_epi2_), the high sensitivity suggested that an interference response from unrelated substances in serum can be negligible.

To further validate the specificity of these peptides in both QD-biosensor or ELISA assays, we test the reactivity between peptides and commercially available anti-S or anti-N antibodies, compared to complete proteins. The results clearly showed that while S protein cross-reacted with anti-HKU1 and anti-MERS-CoV S antibodies (Figure S3a,b, black bar), neither S_epi1_ (Figure S3a,b, red bar) nor S_epi2_ (Figure S3a,b, green bar) cross-reacted with anti-S antibodies of these two coronaviruses. Similarly, N protein cross-reacted with anti-HKU1 and anti-NL63 N antibodies (Figure S3d,e, black bar), but neither N_epi1_ (Figure S3d,e, red bar) nor N_epi2_ (Figure S3d,e, green bar) cross-reacted with these antibodies. Even at lower antibody dilutions (1:500), QD-peptides exhibited no cross-reaction with other coronavirus antibodies (Figure S3c,f). The results demonstrated that the fluorescent QD-peptides biosensor possessed high specificity to avoid cross-reactivity (Figure S3).

### 3.5. Fluorescent QD-Peptides Biosensors Exhibit Excellent Clinical Sensitivity

**Figure 5:**
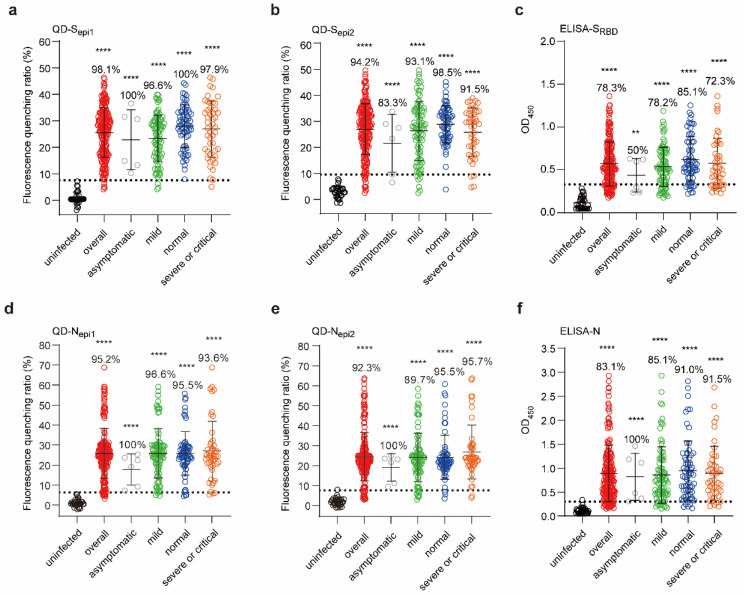
Fluorescent QD-peptides biosensors exhibit excellent clinical sensitivity. (**a**,**b**,**d**,**e**) The reactions of QDs coupled with S_epi1_ (**a**), S_epi2_ (**b**), N_epi1_ (**d**), and N_epi2_ (**e**) to the 207 COVID-19 convalescents serum samples and 32 uninfected human serum samples. (**c**,**f**) The reactions of S_RBD_ and N proteins with the 207 COVID-19 convalescents serum samples (overall, red) and 32 healthy human serum samples (black) by ELISAs. All serum samples were diluted at 1:2000. The convalescents were classified as asymptomatic (grey, *n* = 6), mild (green, *n* = 87), normal (blue, *n* = 67), severe or critical (orange, *n* = 47) according to the illness severity. The horizontal dotted lines in (**a**–**f**) indicated the cutoff value was the mean value +3 SD of uninfected human serum samples. The percentage is the positive detection rate. Dots represented the average of two or three independent measurements. Lines and error bars indicate the mean and SD of each group, respectively. Asterisks (*) indicate significance when the indicated group compared with the uninfected group and were carried out with the unpaired *t*-tests for each experiment (** *p* < 0.01, **** *p* < 0.0001).

To validate the clinical sensitivity of the fluorescent QD-peptides biosensor, we tested 207 COVID-19 convalescents serum samples (infected by Wuhan strain) with different illness severity and 32 uninfected individuals’ serum samples [27] at 1:2000 serum dilutions. The results showed that the QD-peptides biosensors achieved higher sensitivity than protein-based ELISA assays. The overall sensitivity (positive rate) for QD-S_epi1_ is 98.1% (Figure 5a, red dots) and 94.2% for QD-S_epi2_ (Figure 5b, red dots), while that for S_RBD_-based ELISA is much lower at 78.3% (Figure 5c, red dots).

Consistent with S peptides, QD-N peptides also showed higher sensitivity. The overall sensitivity for QD-N_epi1_ is 95.2% (Figure 5d, red dots) and 92.3% for QD-N_epi2_ (Figure 5e, red dots), while that for N-based ELISA is only 83.1% (Figure 5f, red dots). Significantly, we noticed that QD-S_epi1_ reached 100% positive in asymptomatic (Figure 5a, grey dots) and normal severity populations (Figure 5a, blue dots), supporting the high sensitivity of the QD-peptides biosensor.

Moreover, fluorescence quenching ratios of QD-peptides biosensors among the 207 samples were very concentrated, while ELISA OD_450_ values were scattered. We, therefore, performed a dispersion coefficient analysis of the above results (Table 1). The lower the dispersion coefficients are, the higher the results’ consistencies are. The dispersion coefficients for QD-S_epi1_ (36.32%) and QD-S_epi2_ (36.42%) were indeed lower than those of ELISA-S_RBD_ (45.24%). Again, the dispersion coefficients for QD-N_epi1_ (48.28%) and QD-N_epi2_ (49.49%) were also lower than thoses of ELISA-N (66.44%). The results indicated that the QD-peptides biosensor showed higher consistency among large populations.

To further assess the detection performance across the different SARS-CoV-2 variants by the fluorescent QD-peptides biosensors, we tested ten serum samples of omicron-infected or delta-infected patients (Figure S4). The results showed that both QD-S peptides and QD-N peptides could recognize serum samples of SARS-CoV-2-variant infections as efficiently as ELISA assay (similar positive rates). However, the difference between positive and negative groups was more significant in the QD-peptides assay (7–18-fold) than in the ELISA assay (3–5-fold). Thus, the QD-peptides biosensor can also be applied to SARS-CoV-2-variants infections.

## 4. Discussion

Since the outbreak of SARS-CoV-2, precise diagnostic tests have been required to control its spread. Here, we developed a new fluorescent QD-peptides biosensor for detecting COVID-19 antibody-positive serum. After QDs were coupled with SARS-CoV-2 peptides, converting a biological signal to an optical signal could be achieved, significantly improving the sensitivity and specificity of the antibody detection. Such a ‘proof of concept’ design of QD-peptides biosensor can be applied to detect antibodies against other pathogens.

Fluorescent QDs have potential application value to diagnose viral infection and can rapidly and sensitively detect antibodies or viral antigens [28,29]. QD-conjugated anti-IgG is used as a secondary antibody to detect anti-ZIKV IgG antibodies in microplates sensitized with ZIKV envelope E proteins [28]. The detection limit is 100-fold higher than that of peptide-based ELISA methods [28]. In another study, an anti-HA antibody is linked to aspartic acid moieties in the linker peptides chain between QDs and AuNPs to form a CdZnSeS/ZnSeS QD-peptides-AuNP biosensor for detecting influenza virus particles, wherein the influenza virus is detected in the wide linear range of 10^−14^ to 10^−9^ g/mL [29]. However, QDs have never been directly conjugated to a SARS-CoV-2 peptide forming biosensors to transform biological signals to optical signals for COVID-19 serum detection. Our study found that QDs coupled with SARS-CoV-2 B-cell epitopes significantly distinguished COVID-19 patients from the uninfected population.

Interestingly, QDs coupled with SARS-CoV-2 proteins had weaker biosensor reactivity than QD-peptides. A possible explanation for this result is that QDs matched with only peptides because peptides’ size, electrochemical property, and structure may perfectly match QDs for the most efficient energy coupling [30]. In contrast, proteins with spatial conformations may elongate the effective distance between QDs and the antibody, reducing the efficiency of ET or FRET [26]. Therefore, our results suggested stringent requirements for energy coupling between the QDs and the unique advantages of peptides over proteins in the fluorescent QDs biosensors.

It is known that antibodies recognize an antigen via binding to an epitope on the protein surface [31]. Thus, epitopes are the most direct and sensitive elements to capture the corresponding antibody. A recent study uses gold nanoparticles linked to SARS-CoV-2 epitopes to detect antibodies in plasma wherein the gold nanoparticles must be aggregated together, bridging by the antibody to achieve signals [32]. By contrast, our QD-peptides can achieve a sensitive signal individually at a single nanoparticle scale. No aggregation or amplification process is needed. Moreover, the gold nanoparticles need to be coupled to biotinylated peptides through streptavidin [32], which is similar to the principle of SA-ELISA, resulting in low sensitivity. However, the surface of QDs is highly elastic that can directly be connected to the amino groups of peptides through carboxyl groups without any linker to ensure high sensitivity.

The current COVID-19 serological test is mainly based on a commercial ELISA kit using S or N proteins to determine SARS-CoV-2 IgG or IgM antibodies, achieving 82.2–100% sensitivity at 1:20–1:100 serum dilutions [33,34,35,36]. However, ELISA significantly costs workflow, time, and specialized personnel [37]. Furthermore, proteins-based ELISA (use complete viral proteins as a bait) always generates cross-reactions to viruses from the same viral family leading to false-positive [38]. By contrast, the fluorescent QD-peptides biosensors could also achieve over 92% sensitivity (vs. 78% sensitivity for ELISA) in authentic COVID-19 serum samples at 1:2000 serum dilution and avoid cross-reactions as much as possible by using SARS-CoV-2 specific B-cells epitopes. Our results are similar to the study by Liu et al. (using overlapping sera) finding that high levels of specific antibodies were present 1 year after SARS-CoV-2 infection [27]. Still, our QD-peptides biosensors (98.1% sensitivity for QD-S_epi1_ at 1:2000 sera dilution) are more sensitive to the ELISA assay (RBD-based) at an even lower sera dilution (1:100) used in the study by Liu et al. [27]. Thus, these comparisons once again prove the high sensitivity and advantage of saving sera material of our technology. Additionally, the fluorescent QD-peptides biosensor improved the antigen-antibody reactions to more rapid (within 5 min), high-throughput, quantitative, and timely clinical detection.

Despite the frequent emergence of SARS-CoV-2 variants, the fluorescent QD-peptides biosensor could perform with high sensitivity in detecting SARS-CoV-2 variant-infected patients’ serum samples. Compared to proteins, peptides are easy to update, produce, and display dominant epitopes when the virus mutates or a new virus emerges. Another advantage is to conjugate multiple peptides into one QD particle, which may provide feasible detection of antibodies against various antigens for further studies.

Moreover, commercial ELISA kits usually use SARS-CoV-2 N and S proteins, making ELISA unable to effectively distinguish SARS-CoV-2 from other human coronaviruses due to protein homology [38,39]. In contrast, our study’s highly selective SARS-CoV-2 peptides showed low homology and limited cross-reaction with other HCoVs (<45%), which can further increase the accuracy of diagnoses. Finally, the specific orbicular surface area of QDs supports multivalent connections for combining different peptides to achieve multiple diagnostics, which is impossible for traditional serum diagnostics. We verified that the combination of S and N peptides provided a higher detection rate by peptides-ELISA (data not shown). In the infected and vaccinated population, the antibody to N proteins is the most abundant, and the antibody to S proteins provides specificity. Thus, combined epitopes are superior for COVID-19 antibody detection. Intriguingly, the S_epi1_ located inside the RBD domain presents neutralizing epitopes so that QD-S_epi1_ might react with neutralizing antibodies for further application.

Currently, the fluorescence signal could be measured by a multifunctional microplate reader for high-throughput connected to a computer. In the future, a particular application will be developed for smartphones to receive results from the multifunctional microplate reader, making serological testing wearable, personal and intelligent. As more of the world’s population is vaccinated and evaluation of the antibody levels becomes an essential indicator for vaccine efficiency, we believe that this QD-peptides biosensor has the potential to be used for such large-scale needs.

Taken together, we, for the first time, developed QDs coupled with SARS-CoV-2 peptides for the detection of COVID-19-positive serum. The optical signal amplifies the biological signal and dramatically improves the detection sensitivity within minutes with a detection limit of 100 pM, making the large-scale onsite diagnosis possible.

## Figures and Tables

**Table 1:** Dispersion coefficient of data values from either QD-peptides or ELISA assay with the indicated antigen.

Severity of Illness	ELISA-S_RBD_	QD-S_epi1_	QD-S_epi2_	ELISA-N	QD-N_epi1_	QD-N_epi2_
Asymptomatic (6)	45.08%	49.47%	51.36%	60.35%	55.19%	62.25%
Mild (87)	42.68%	37.97%	43.46%	69.12%	47.76%	50.63%
Normal (67)	42.34%	28.86%	24.92%	66.34%	42.00%	45.49%
Severe or critical (47)	50.35%	39.51%	35.95%	63.42%	55.21%	50.30%
Overall (207)	45.24%	36.32%	36.42%	66.44%	48.28%	49.49%

## Data Availability

All data needed to evaluate the conclusions in the paper are present in the paper and/or the Supplementary Materials.

## Supplementary Materials

The following supporting information can be downloaded at: https://www.mdpi.com/article/10.3390/v14051031/s1, Figure S1. Epitope prediction of the SARS-CoV-2 S proteins; Figure S2. Epitope prediction of the SARS-CoV-2 N proteins; Figure S3. The specificity of the fluorescent QD-peptides biosensors; Figure S4. The sensitivity across the different SARS-CoV-2 variants by the fluorescent QD-peptides biosensor; Table S1. Homology analysis of the four peptides; Table S2. Epitope prediction of SARS-CoV-2 S proteins by DiscoTope 2.0 method; Table S3. Epitope prediction of SARS-CoV-2 S proteins by Bepipred Linear Epitope Prediction 2.0 method; Table S4. Epitope prediction of SARS-CoV-2 N proteins by Bepipred Linear Epitope Prediction 2.0 method; Table S5. Epitope prediction of SARS-CoV-2 S proteins by Emini Surface Accessibility Prediction method; Table S6. Epitope prediction of SARS-CoV-2 N proteins by Emini Surface Accessibility Prediction; Table S7. The clinical records of the SARS-CoV-2 convalescents; Table S8. The clinical records of the SARS-CoV-2 variant-infected patients.
