# Supplementary material for: B-Cell-Epitope-Based Fluorescent Quantum Dot Biosensors for SARS-CoV-2 Enable Highly Sensitive COVID-19 Antibody Detection
Source: Viruses. 2022 May 12;14(5):1031. doi: 10.3390/v14051031 (PMC9145955; doi:10.3390/v14051031)
Supplement: Supplementary file 1 [file viruses-14-01031-s001.zip › viruses-1602857-supplementary.pdf]

a

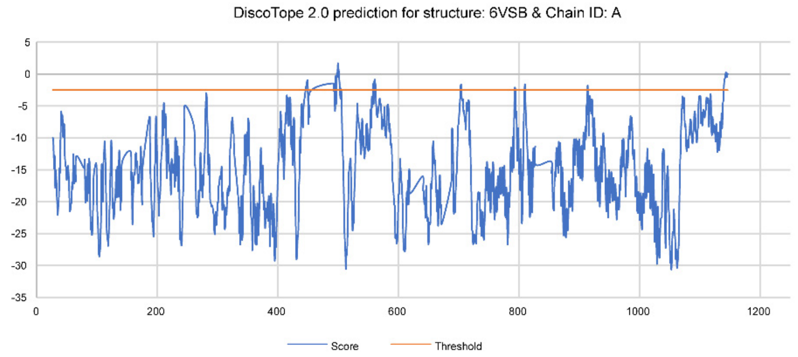

b

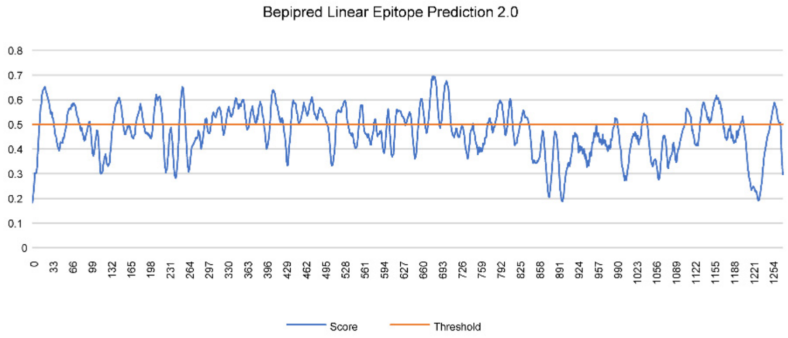

c

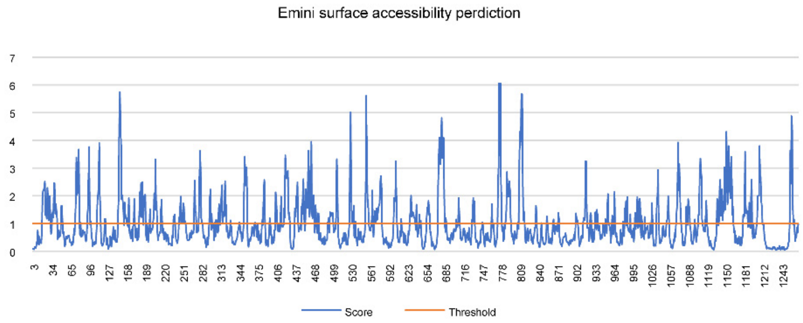

Figure S1. Epitope prediction of the SARS-CoV-2 S proteins. (**a**) Predict conformational B cell epitopes on SARS-CoV-2 S proteins structure (PDB:6VSB) with the DiscoTope 2.0 and a cutoff of ≥ -2.5. (**b**) Predict linear B cell epitope on S proteins (NCBI: YP_009724390.1) with BepiPred 2.0 and a cutoff of ≥ 0.5. (**c**) Predict linear B cell epitopes on the S proteins (NCBI: YP_009724390.1) with Emini Surface Accessibility Prediction and a cutoff of ≥ 1.0.

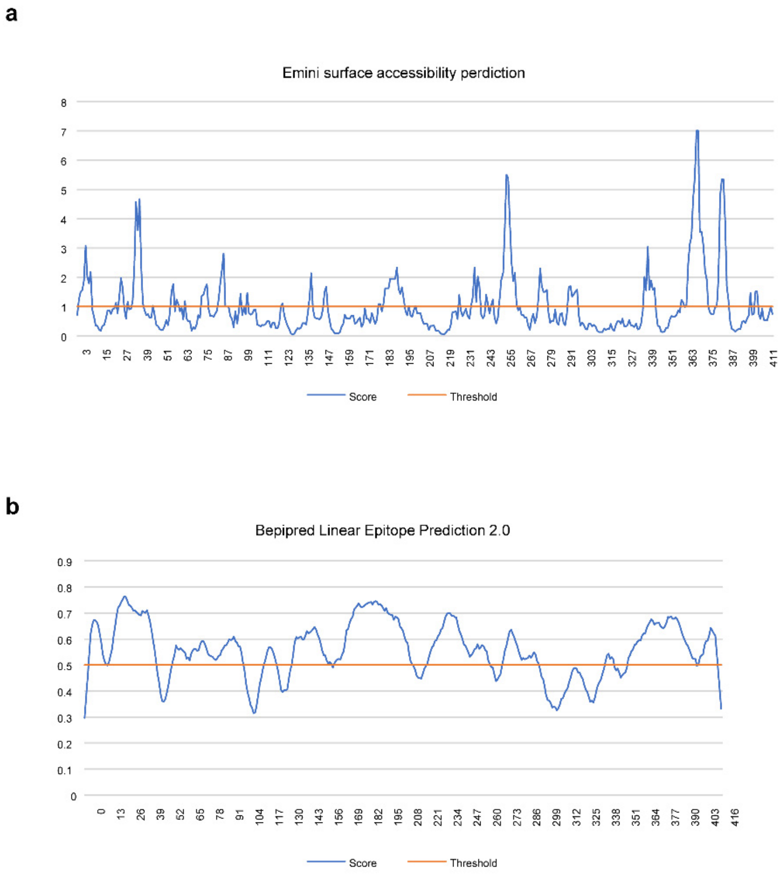

Figure S2. Epitope prediction of the SARS-CoV-2 N proteins. (**a**) Predict linear B cell epitope on N proteins (NCBI: YP_009724397.2) with BepiPred 2.0 and a cutoff of ≥ 0.5. (**b**) Predict linear B cell epitopes on N proteins (NCBI: YP_009724397.2) with Emini Surface Accessibility Prediction and a cutoff of ≥ 1.0.

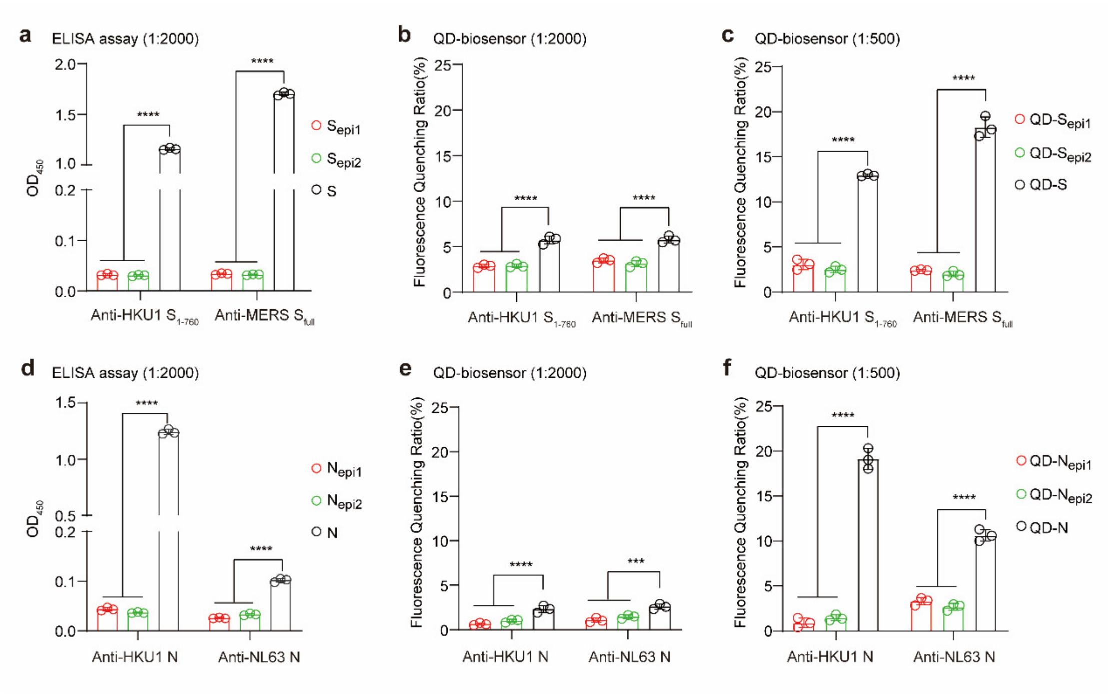

Figure S3. The specificity of the fluorescent QD-peptides biosensors. (**a**) The reactions of S peptides (S_{full} proteins as a control) and commercial anti-HKU1 S_{1-760} or anti-MERS S_{full} polyclonal antibody diluted at 1:2000 by ELISAs. (**b-c**) The reactions of QD-S peptides (S_{full} proteins as a control) and commercial anti-HKU1 S_{1-760} or anti-MERS S_{full} polyclonal antibody diluted at 1:500 (**c**) or 1:2000 (**b**). (**d**). The reactions of N peptides (N proteins as a control) and commercial anti-HKU1 or anti-NL63 N polyclonal antibody diluted at 1:2000 by ELISAs. (**e-f**) The reaction of QD-N peptides (N proteins as a control) and commercial anti-HKU1 or anti-NL63 N polyclonal antibodies diluted at 1:500 (**f**) or 1:2000 (**e**). Data were presented as mean ± SD of three independent measurements and were carried out with unpaired t-tests for each experiment (***p<0.001, ****p<0.0001).

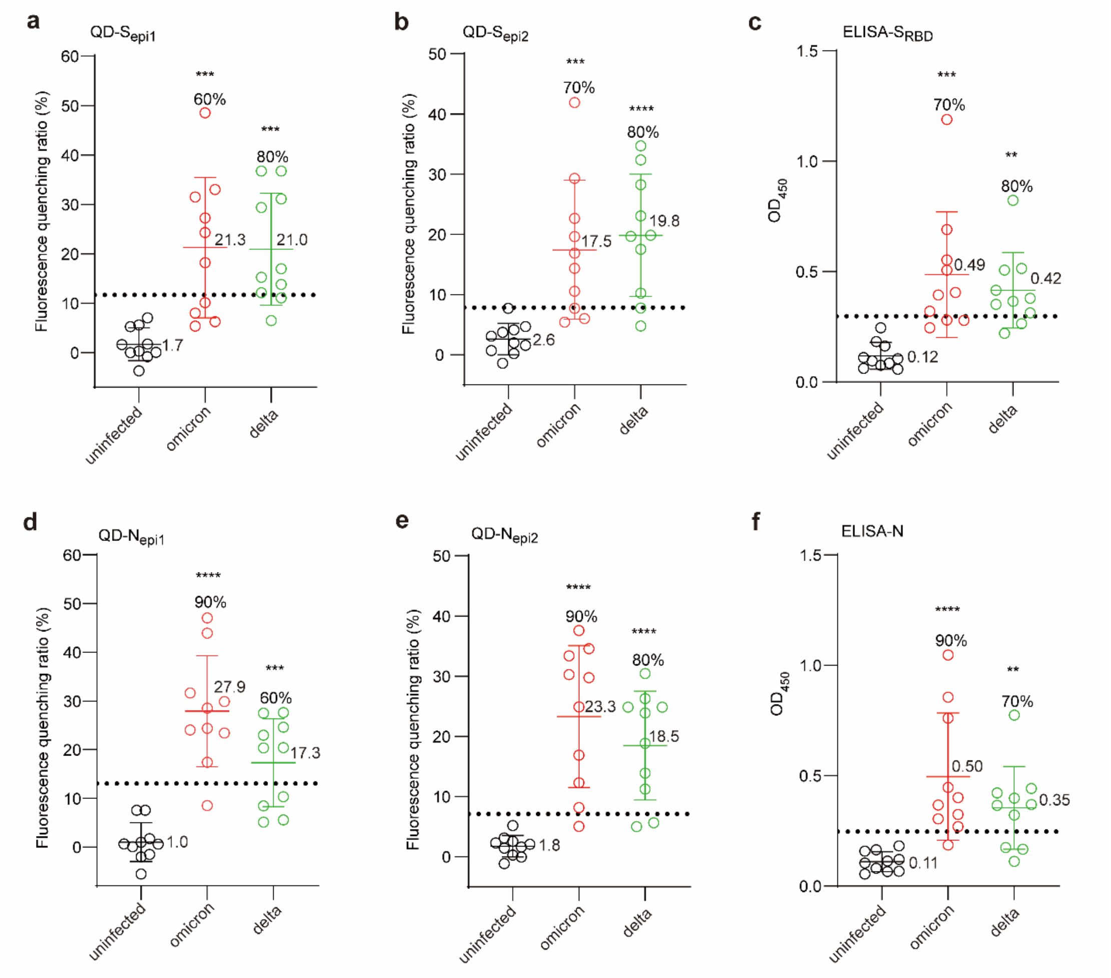

Figure S4. The sensitivity across the different SARS-CoV-2 variants by the fluorescent QD-peptides biosensor. (**a**-**b** and **d**-**e**) The reactions of QD-S_{epi1} (**a**), QD-S_{epi2} (**b**), QD-N_{epi1} (**d**), and QD-N_{epi2} (**e**) to 10 SARS-CoV-2 omicron-infected patients' serum samples (red) or 10 SARS-CoV-2 delta-infected patients' serum samples (green). (**c** and **f**) The reaction of S_{RBD} (**c**) and N (**f**) to SARS-CoV-2 omicron-infected patients serum samples (red) or 10 SARS-CoV-2 delta-infected patient serum samples (green) by ELISAs. All serum samples were diluted at 1:200. The horizontal dotted lines in (**a**-**f**) indicated the cutoff value was the mean value +3 SD of uninfected human serum samples. The percentage is the positive detection rate. Each group is marked with the mean value. Dots represented the average of two or three independent measurements. Lines and error bars indicate the mean and SD of each group, respectively. Asterisks (*) indicate significance when the indicated group compared with the unfaired t-tests for each experiment (**$p<0.01$, ***$p<0.001$, ****$p<0.0001$).

Table S1. Homology analysis of the four peptides.

**S_{epi1}**		
Virus	Amino acid sequence	Homology (%)
SARS-CoV-2	PLQSYGFQPTNGVGY	100
SARS-CoV	PLNDYGFYTTTGIGY	60
MERS-CoV	PLEGGGWLVASGSTV	27
HCoV-HKU1	PRSCSQKKSLVGVGE	27
HCoV-OC43	TYKCPQSKSLVGIGE	13
HCoV-229E	PFSFGKVNNFVKFGS	13
HCoV-NL63	PFSFSKLNNFQKFKT	7
**S_{epi2}**		
Virus	Amino acid sequence	Homology (%)
SARS-CoV-2	KQIYKTPPIKD	100
SARS-CoV	KQMYKTPTLKY	64
MERS-CoV	KSSQSSPIIPGF	9
HCoV-HKU1	MQGVTLSSNLNT	9
HCoV-OC43	MNGVTLSTKLKD	18
HCoV-229E	DKKAFTLANVSS	0
HCoV-NL63	DSNAFSLANVTS	0
**N_{epi1}**		
Virus	Amino acid sequence	Homology (%)
SARS-CoV-2	EPKKDKKKKAD	100
SARS-CoV	EPKKDKKKKTD	91
MERS-CoV	EKKQKAPKEE	45
HCoV-HKU1	TVSGSLSPKPQ	9
HCoV-OC43	DGMMNMSPKPQ	9
HCoV-229E	LLNPSALEFNP	0
HCoV-NL63	SQSSHVAQNTV	0
**N_{epi2}**		
Virus	Amino acid sequence	Homology (%)
SARS-CoV-2	QALPQRQKKQ	100
SARS-CoV	QPLPQRQKKQP	82
MERS-CoV	DQMSEPPKEQR	9
HCoV-HKU1	RGVKQLPEQFD	9
HCoV-OC43	RGHKNGQGEND	9
HCoV-229E	TSPATAEPVRD	0
HCoV-NL63	ACTPESKPLAD	0

Table S2. Epitope prediction of SARS-CoV-2 S proteins by DiscoTope 2.0 method.

NO.	Start Position	Amino acid sequence	End Position
1	491	PLQSYGFQPTNGVGY	505
2	558	KFLPF	562
3	1140	PLQPELD	1146

Table S3. Epitope prediction of SARS-CoV-2 S proteins by Bepipred Linear Epitope Prediction 2.0 method.

NO.	Start Position	Amino acid sequence	End Position
1	13	SQCVNLTTRTQLPPAYTNSFTRGVY	37
2	59	FSNVTWFHAIHVSGTNGTKRFDN	81
3	138	DPFLGVYYHKNNKSWME	154
4	177	MDLEGKQGNFKNL	189
5	206	KHTPINLVRDLPQGFS	221
6	250	TPGDSSSGWTA	260
7	304	KSFTVEKGIYQTSNFRVQP	322
8	329	FPNITNLCPFGEVFNATRFASVYAWNRKRISNCVA	363
9	369	YNSASFSTFKCYGVSPTKLNDLCFT	393
10	404	GDEVRQIAPGQTGKIADYNYKLP	426
11	440	NLDSKVGGNYNYLYRLFRKSNLKPFERDISTEIYQAGSTP CNGVEGFNCYFPLQSYGFQPTN	501
12	516	ELLHAPATVCGPKKSTNLVKN	536
13	555	SNKKFLPF	562
14	602	TNTSN	606
15	616	NCTEVPVAIHADQLTPT	632
16	634	RVYSTGSNVFQ	644
17	656	VNNSYECDIPI	666
18	672	ASYQTQTNSPRRARSVASQ	690
19	695	YTMSLGAENSVAYSNN	710
20	773	EQDKNTQ	779
21	786	KQIYKTPPIKDFGGF	800
22	807	PDPSKPSK	814
23	828	LADAGFIKQYGDCLG	842
24	988	EAEVQ	992
25	1035	GQSKRVDFC	1043
26	1107	RNFYEPQIITTD	1118
27	1133	VNNTVYDPLQPELDSFKEELDKYFKNHTSPDVDLGDISGI	1172
28	1252	SCCKFDEDDSEPVLKG	1267

Table S4. Epitope prediction of SARS-CoV-2 N proteins by Bepipred Linear Epitope Prediction 2.0 method.

NO.	Start Position	Amino acid sequence	End Position
1	4	NGPQNQRNAPRI	15
2	17	FGGPSDSTGSNQNGERSGARSKQRRPQ GLPNN	48
3	59	HGKEDLKFPRGQGVPINTNSSPDDQIG YYRRATRRIRGGDGKMKDLS	105
4	119	AGLPYGANK	127
5	137	GALNTPKDHIGTRNPANNAAIVLQLPQ	163
6	165	TTLPKGFYAEGSRGGSQASSRSSSRSRN SSRNSTPGSSRGTSPARMAGNGGD	216
7	226	RLNQLESKMSGKGQQQQGQTVTKKSA AEASKKPRQKRTATKA	267
8	276	RRGPEQTQGNFGDQELIRQGTDYK	299
9	343	DPNFKD	348
10	358	DAYKTFPPTEPKKDKKKKADETQALP QRQKKQQTVTLLPAADLDD	402
11	404	SKQLQQSMSSADS	416

Table S5. Epitope prediction of SARS-CoV-2 S proteins by Emini Surface Accessibility Prediction method.

NO.	Start Position	Amino acid sequence	End Position
1	23	QLPPAYTNSFTRGVYYPD	40
2	74	NGTKRFDNPVL	84
3	143	VYYHKNNKSWMESEFR	158
4	183	QGNFKNL	189
5	272	PRTFLLKYNE	281
6	314	QTSNFRVQPTE	324
7	415	TGKIADYNYK	424
8	442	DSKVGGNYNYLYRLFRKSNLKPFERDI STEI	472
9	498	QPTNGVG	504
10	556	NKKFLPFQQF	565
11	570	ADTTDAVRDP	579
12	675	QTQTNSPRRARS	686
13	777	NTQEVFAQVK	786
14	788	IYKTPPI	794
15	806	LPDPSKPSKRSFI	818
16	919	NQKLIA	924
17	1139	DPLQPELDSFKEELDKYFKNHTSPDV	1164
18	1184	DRLNEVAKN	1192
19	1204	GKYEQYIKW	1212

Table S6. Epitope prediction of SARS-CoV-2 N proteins by Emini Surface Accessibility Prediction.

NO.	Start Position	Amino acid sequence	End Position
1	4	NGPQNQRN	11
2	36	RSKQRRP	42
3	87	YRRATR	92
4	185	RSSSRSRNSSRNS	197
5	237	KGQQQQ	242
6	254	ASKKPRQKRTA	264
7	277	RGPEQT	282
8	295	GTDYKH	300
9	340	DDKDPNF	346
10	365	PTEPKKDKKKKAD	377
11	384	QRQKKQQ	390

Table S7. The clinical records of the SARS-CoV-2 convalescents.

Sample number	Gender	Age	Illness severity	Date of symptom onset	Date of sample collection
1	male	37	critical	2020/2/9	2021/1/19
2	female	43	mild	2020/2/14	2021/1/19
3	male	56	mild	2020/2/5	2021/1/19
4	female	78	mild	2020/2/26	2021/1/19
5	female	73	asymptomatic	2020/2/14	2021/1/19
6	female	67	mild	2020/1/23	2021/1/19
7	male	51	mild	2020/2/5	2021/1/19
8	female	66	mild	2020/2/10	2021/1/19
9	female	51	mild	2020/2/15	2021/1/19
10	male	78	normal	2020/2/12	2021/1/19
11	male	39	mild	2020/2/12	2021/1/19
12	female	76	mild	2020/2/1	2021/1/19
13	female	51	normal	2020/2/10	2021/1/19
14	male	69	mild	2020/2/14	2021/1/19
15	male	59	normal	2020/2/7	2021/1/19
16	female	70	mild	2020/2/5	2021/1/19
17	female	56	severe	2020/2/5	2021/1/19
18	male	63	mild	2020/2/7	2021/1/19
19	female	68	severe	2020/1/26	2021/1/19
20	female	35	mild	2020/2/18	2021/1/19
21	female	60	mild	2020/2/12	2021/1/19
22	male	66	severe	2020/1/31	2021/1/19
23	male	79	mild	2020/1/25	2021/1/19
24	female	61	mild	2020/2/18	2021/1/19
25	male	36	mild	2020/2/11	2021/1/19
26	female	70	mild	2020/1/31	2021/1/19
27	female	64	normal	2020/2/10	2021/1/19
28	female	63	mild	2020/2/5	2021/1/19
29	female	62	asymptomatic	2020/2/28	2021/1/19
30	male	68	severe	2020/2/7	2021/1/19
31	male	64	severe	2020/2/3	2021/1/19
32	female	66	mild	2020/1/23	2021/1/19
33	male	67	normal	2020/1/21	2021/1/19
34	female	61	normal	2020/1/28	2021/1/19
35	male	66	severe	2020/2/4	2021/1/19
36	female	73	mild	2020/2/28	2021/1/19
37	female	59	normal	2020/1/27	2021/1/19
38	female	73	normal	2020/2/12	2021/1/19
39	female	60	severe	2020/1/31	2021/1/19
40	female	54	mild	2020/1/26	2021/1/19

41	male	65	severe	2020/1/25	2021/1/19
42	male	76	severe	2020/1/30	2021/1/19
43	female	59	normal	2020/2/2	2021/1/19
44	male	57	normal	2020/2/2	2021/1/19
45	female	58	mild	2020/1/23	2021/1/19
46	female	50	normal	2020/1/21	2021/1/19
47	female	47	severe	2020/1/30	2021/1/19
48	male	58	severe	2020/2/11	2021/1/19
49	female	67	mild	2020/2/5	2021/1/19
50	female	65	severe	2020/2/3	2021/1/19
51	female	43	normal	2020/1/28	2021/1/19
52	male	59	mild	2020/1/27	2021/1/19
53	male	66	normal	2020/2/13	2021/1/19
54	female	62	critical	2020/1/31	2021/1/19
55	male	57	severe	2020/1/31	2021/1/19
56	female	61	normal	2020/2/16	2021/1/19
57	female	61	mild	2020/2/18	2021/1/19
58	female	61	severe	2020/1/12	2021/1/19
59	female	59	normal	2020/2/4	2021/1/19
60	female	59	severe	2020/1/26	2021/1/19
61	male	76	mild	2020/2/12	2021/1/19
62	male	34	normal	2020/1/30	2021/1/19
63	male	72	normal	2020/1/22	2021/1/19
64	female	74	normal	2020/1/27	2021/1/19
65	female	44	normal	2020/1/20	2021/1/19
66	male	56	severe	2020/1/31	2021/1/19
67	female	69	mild	2020/2/1	2021/1/19
68	male	52	mild	2020/2/6	2021/1/19
69	female	57	normal	2020/2/13	2021/1/19
70	female	66	mild	2020/2/1	2021/1/19
71	female	62	mild	2020/2/2	2021/1/19
72	female	56	normal	2020/2/6	2021/1/19
73	male	48	mild	2020/1/28	2021/1/19
74	female	45	mild	2020/1/22	2021/1/19
75	female	52	mild	2020/1/29	2021/1/19
76	female	50	normal	2020/2/1	2021/1/19
77	male	58	mild	2020/2/20	2021/1/19
78	female	56	critical	2020/1/23	2021/1/19
79	female	51	mild	2020/2/1	2021/1/19
80	male	57	normal	2020/2/1	2021/1/19
81	male	55	severe	2020/2/4	2021/1/19
82	female	53	normal	2020/2/3	2021/1/19
83	male	26	mild	2020/1/31	2021/1/19
84	male	58	severe	2020/1/26	2021/1/19
85	female	64	mild	2020/2/13	2021/1/19
86	male	44	normal	2020/1/28	2021/1/19
87	female	45	normal	2020/2/10	2021/1/19
88	female	55	asymptomatic	2020/3/6	2021/1/19
89	female	54	normal	2020/2/17	2021/1/23
90	male	40	critical	2020/1/23	2021/1/22
91	female	42	normal	2020/2/20	2021/1/23
92	male	68	mild	2020/1/13	2021/1/22
93	male	23	mild	2020/1/29	2021/1/23
94	male	64	mild	2020/2/16	2021/1/22
95	female	67	normal	2020/1/23	2021/1/22
96	female	39	severe	2020/2/18	2021/1/23
97	male	56	mild	2020/2/6	2021/1/22
98	female	28	mild	2020/2/9	2021/1/23
99	male	56	severe	2020/2/7	2021/1/26
100	male	57	mild	2020/2/10	2021/1/23
101	female	68	mild	2020/2/15	2021/1/23
102	female	37	mild	2020/2/2	2021/1/26
103	female	68	severe	2020/2/11	2021/1/22
104	female	54	normal	2020/1/15	2021/1/23

105	female	29	mild	2020/2/20	2021/1/23
106	female	54	normal	2020/2/16	2021/1/22
107	male	57	mild	2020/2/10	2021/1/22
108	male	63	normal	2020/2/9	2021/1/22
109	male	61	normal	2020/1/30	2021/1/23
110	female	69	normal	2020/2/17	2021/1/22
111	male	61	severe	2020/2/8	2021/1/22
112	female	70	mild	2020/2/20	2021/1/22
113	male	72	mild	2020/2/6	2021/1/22
114	male	50	normal	2020/1/24	2021/1/22
115	female	59	mild	2020/2/9	2021/1/23
116	male	46	mild	2020/2/18	2021/1/23
117	female	57	normal	2020/1/28	2021/1/26
118	female	69	mild	2020/2/7	2021/1/23
119	male	33	mild	2020/2/18	2021/1/23
120	male	64	normal	2020/2/27	2021/1/23
121	male	36	normal	2020/1/24	2021/1/22
122	female	40	normal	2020/2/18	2021/1/22
123	female	67	mild	2020/2/10	2021/1/22
124	male	62	mild	2020/2/12	2021/1/23
125	female	57	mild	2020/2/18	2021/1/26
126	female	67	severe	2020/2/18	2021/1/26
127	female	55	mild	2020/1/26	2021/1/26
128	female	52	normal	2020/2/6	2021/1/23
129	female	30	mild	2020/3/6	2021/1/23
130	male	42	severe	2020/2/18	2021/1/26
131	male	64	mild	2020/2/9	2021/1/23
132	male	62	asymptomatic	2020/3/1	2021/1/22
133	male	33	normal	2020/2/9	2021/1/22
134	female	44	normal	2020/2/4	2021/1/22
135	female	26	mild	2020/2/7	2021/1/23
136	male	32	mild	2020/2/8	2021/1/22
137	female	59	mild	2020/2/12	2021/1/22
138	female	58	normal	2020/2/10	2021/1/23
139	male	83	mild	2020/2/10	2021.1.20
140	female	78	normal	2020/2/10	2021.1.20
141	female	74	mild	2020/2/14	2021.1.20
142	female	65	normal	2020/2/11	2021.1.20
143	male	71	severe	2020/1/31	2021.1.20
144	female	74	critical	2020/1/18	2021.1.20
145	female	62	severe	2020/1/21	2021.1.20
146	female	46	critical	2020/1/31	2021.1.20
147	female	40	mild	2020/2/14	2021.1.20
148	male	44	severe	2020/2/1	2021.1.20
149	male	76	normal	2020/2/3	2021.1.20
150	male	69	mild	2020/2/2	2021.1.20
151	male	69	mild	2020/1/22	2021.1.20
152	female	68	mild	2020/1/22	2021.1.20
153	female	70	mild	2020/2/6	2021.1.20
154	female	72	mild	2020/1/27	2021.1.20
155	male	33	mild	2020/1/25	2021.1.20
156	female	59	normal	2020/1/26	2021.1.20
157	female	64	normal	2020/2/5	2021.1.20
158	female	87	normal	2020/2/8	2021.1.20
159	male	50	mild	2020/2/11	2021/1/23
160	male	65	normal	2020/1/1	2021/1/22
161	female	66	normal	2020/1/30	2021/1/26
162	male	63	severe	2020/2/7	2021/1/22
163	female	60	normal	2020/1/25	2021/1/26
164	male	62	critical	2020/2/6	2021/1/22
165	male	52	normal	2020/2/13	2021/1/22
166	female	51	mild	2020/2/9	2021/1/23
167	male	30	normal	2020/2/6	2021/1/23
168	male	72	mild	2020/1/16	2021/1/26

169	female	67	severe	2020/2/6	2021/1/22
170	female	58	normal	2020/2/18	2021/1/26
171	female	27	normal	2020/2/20	2021/1/22
172	male	63	severe	2020/1/26	2021/1/22
173	male	64	severe	2020/2/3	2021/1/22
174	female	63	mild	2020/2/2	2021/1/22
175	female	63	critical	2020/1/19	2021/1/22
176	male	32	severe	2020/1/22	2021/1/22
177	female	63	critical	2020/1/27	2021/1/22
178	male	67	normal	2020/2/6	2021/1/22
179	male	68	severe	2020/2/3	2021/1/22
180	female	61	severe	2020/2/6	2021/1/22
181	female	36	normal	2020/2/13	2021/1/22
182	male	56	asymptomatic	2020/3/24	2021/1/22
183	female	64	normal	2020/2/2	2021/1/22
184	female	66	mild	2020/1/21	2021/1/22
185	male	67	critical	2020/1/31	2021/1/22
186	male	67	severe	2020/2/2	2021/1/22
187	female	66	normal	2020/1/21	2021/1/22
188	male	66	normal	2020/2/6	2021/1/22
189	male	67	normal	2020/1/31	2021/1/22
190	male	68	normal	2020/2/15	2021/1/22
191	male	74	severe	2020/2/15	2021/1/22
192	female	70	normal	2020/2/2	2021/1/22
193	female	68	mild	2020/2/15	2021/1/22
194	male	66	severe	2020/1/24	2021/1/22
195	male	57	asymptomatic	2020/3/25	2021/1/22
196	male	33	normal	2020/1/29	2021/1/22
197	female	59	mild	2020/2/16	2020/1/23
198	male	50	normal	2020/2/13	2020/1/23
199	male	46	mild	2020/2/13	2020/1/23
200	male	74	mild	2020/1/31	2020/1/23
201	female	72	mild	2020/2/2	2021/1/23
202	female	26	mild	2020/2/26	2021/1/23
203	male	72	mild	2020/2/18	2021/1/23
204	male	61	mild	2020/1/17	2021/1/23
205	female	61	mild	2020/1/21	2021/1/23
206	male	64	normal	2020/2/10	2021/1/26
207	female	71	mild	2020/2/12	2021/1/22

Table S8. The clinical records of the SARS-CoV-2 variant-infected patients.

Sample number	Gender	Age	Illness severity	Date of sample collection
		Omicron		
1	female	36	mild	2022/2/22
2	female	39	normal	2022/2/22
3	female	36	asymptomatic	2022/2/22
4	male	68	normal	2022/2/22
5	male	36	mild	2022/2/22
6	female	43	asymptomatic	2022/2/22
7	male	34	mild	2022/2/22
8	male	41	asymptomatic	2022/2/22
9	male	41	normal	2022/2/23
10	female	40	normal	2022/2/23
		Delta		
1	female	89	normal	2021/8/12
2	female	1	asymptomatic	2021/8/12
3	female	55	normal	2021/8/12
4	male	63	mild	2021/8/12
5	female	27	mild	2021/8/12

6	male	29	mild	2021/8/12
7	male	50	asymptomatic	2021/8/12
8	male	31	normal	2021/8/12
9	male	58	normal	2021/8/12
10	male	55	asymptomatic	2021/8/12
